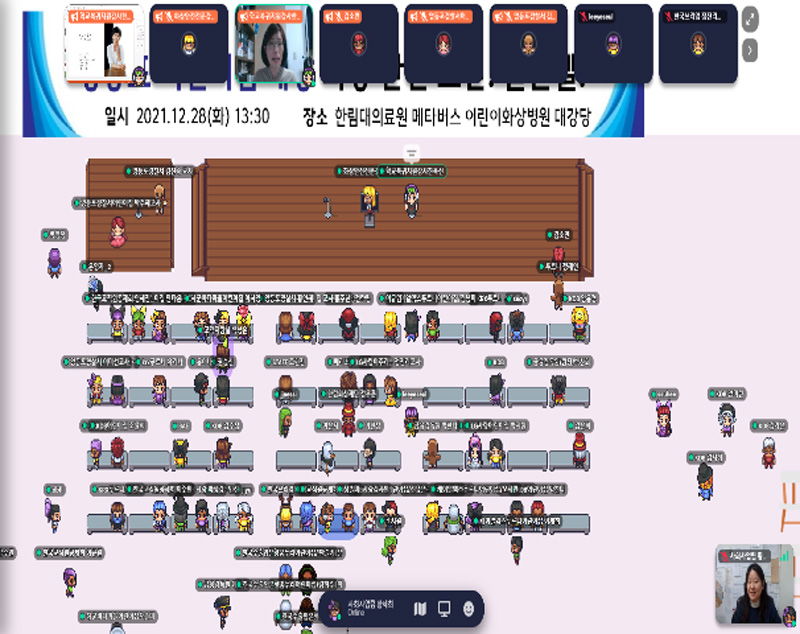# 544 Creating Communities for Burn Survivors Using Metaverse Platform

**DOI:** 10.1093/jbcr/irad045.141

**Published:** 2023-05-15

**Authors:** Se-Hee Hwang

**Affiliations:** Hangang Sacred Heart Hospital of Hallym University Medical Center, SEOUL, Seoul-t'ukpyolsi

## Abstract

**Introduction:**

In the post-COVID-19 era, as face-to-face activities become difficult, the psychosocial difficulties of burn survivors are increasing, and their social isolation is also intensifying. At this time, studies on the effectiveness of online intervention have been reported, and demand for the use of metaverse platforms that can supplement face-to-face activities is also increasing. Accordingly, the metaverse platform was intended to be used to prevent social isolation of burn survivors and to prepare a social support system, and their experiences were analyzed and its impact was investigated.

**Methods:**

Among the various metaverse platforms, Burn Hospital was established on Gather Town platform, which can present or communicate on an online map and has the function of producing or sharing videos, writings, and paintings. Main plaza, auditorium, education room, exhibition room, counseling room, and play room were built on the platform, and burn survivors could access the treatment process and various information, and at the same time, they could easily access the content with their families through programs by treatment stage, age, and group. In particular, various education, workshops, and support groups were held to share burn experiences with each other and provide opportunities to benchmark each other's overcoming strategies. In addition, using Ifland, a mobile phone app-based platform, education on safety and burn awareness improvement campaigns were conducted to citizens.

**Results:**

A total of 700 people participated in a total of 20 education and lectures held at Burn Hospital on Gather Town platform, which opened on December 18, 2021. In addition, art therapy programs for children burn survivors are underway every week. Most of the participants evaluated their experiences on the metaverse platform as new and fun, and it was positively evaluated that geographical limitations could be overcome and community support group meetings were possible.

**Conclusions:**

It was evaluated that the establishment of communities using metaverse has a positive effect on helping the psychosocial recovery of burn survivors and their families and establishing a social support system. In particular, compared to face-to-face services, it was time-wise and economically efficient and had the advantage of being able to experience interesting factors. On the other hand, it is somewhat difficult for burn survivors who have difficulty using PC to access the platform, so digital literacy education should be preceded, and support for improving digital accessibility is needed.

**Applicability of Research to Practice:**

Along with face-to-face services, it is expected that the community activities of burn survivors using the metaverse platform will continue to expand, and it is expected to lead to the development of digital treatments in the future by developing various digital-based clinical programs to help burn survivors to recover physically and socially.